# Positive Response and Increase in ADAMTS13 with Scheduled Rituximab in a Patient with Relapsing Thrombotic Thrombocytopenic Purpura

**DOI:** 10.7759/cureus.5054

**Published:** 2019-07-01

**Authors:** Bahaa Amer, Anjan Patel

**Affiliations:** 1 Internal Medicine, Florida State University College of Medicine, Sarasota, USA; 2 Hematology / Oncology, Florida Cancer Specialists, Sarasota, USA

**Keywords:** thrombotic thrombocytopenic purpura (ttp), rituximab, adamts-13, plasma exchange, altered mental status, acute kidney injury, refractory, relapse, b cell, von willebrand

## Abstract

Thrombotic thrombocytopenic purpura (TTP) is a coagulation disorder caused by a deficiency in ADAMTS13. Patients classically present with symptoms of end-organ damage as well as anemia and thrombocytopenia. Treatment is therapeutic plasma exchange (TPE) in the acute setting, with systemic immunosuppression for refractory cases.

A 48-year-old female diagnosed with TTP at age 42 presented initially with altered mental status (AMS), severe anemia, and thrombocytopenia requiring intensive care unit (ICU) admission. The patient was treated acutely and discharged from the hospital. During subsequent years, multiple relapses requiring hospitalization prompted scheduled maintenance with rituximab. Since maintenance therapy, the patient remained relapse-free while ADAMTS13 levels escalated.

Untreated, TTP is fatal. The treatment goal in the acute setting is the repletion of ADAMTS13 coupled with immunosuppression in refractory cases. Rituximab typically is reserved for patients who do not improve with initial TPE. Albeit unusual in TTP, rituximab maintenance in our patient induced remission.

Maintenance therapy with rituximab in patients with a history of relapsing TTP can blunt or obviate the frequency of relapses and hospital admissions. More research is required to establish the effectiveness of rituximab in the chronic treatment of TTP.

## Introduction

Thrombotic thrombocytopenic purpura (TTP) is rare, dangerous, and characterized by anemia, thrombocytopenia, and hypercoagulability. Etiology is due to ADAMTS13 deficiency, which is caused usually by an acquired antibody. In very rare cases, an ADAMTS13 deficiency is inherited in an autosomal recessive pattern, such as in Upshaw-Schulman syndrome. ADAMTS13 is a protein that naturally cleaves Von Willebrand factor (vWF) multimers to prevent spontaneous coagulation. When these molecules are not properly cleaved, unprompted platelet aggregation ensues, activating the coagulation cascade and resulting in platelet consumption. While vWF molecules cause the shearing of red blood cells, evoking thrombocytopenia and hemolytic anemia. The clots formed by this process can cause emboli, which can block blood vessels and lead to end-organ damage [1].

The classic pentad presentation of thrombocytopenia, hemolytic anemia, fever, reduced kidney function, and altered mental status occurs in a small fraction of patients [1]. Patients more commonly express flu-like symptoms with a wide spectrum of severity. While some may have mild symptoms, others can present with altered mental status (AMS), symptoms of stroke, seizure, or ischemia of various organs, requiring intensive care. Although TTP consumes platelets, massive bleeding is extremely rare; bleeding usually manifests as purpura, ecchymosis, or mucosal bleeding.

Diagnosis is driven by clinical presentation and presumption. A constellation of hemolytic anemia, thrombocytopenia, AMS, and kidney injury (AKI) can be present in diseases such as hemolytic uremic syndrome (HUS). Disseminated intravascular coagulation (DIC) is also a differential diagnosis of TTP. All three differ in certain laboratory values such as PT, PTT, and fibrinogen levels. In institutions where the assay is available, ADAMTS13 can be directly measured; a level of <10% is highly suggestive of TTP while levels >10% suggest a different diagnosis. Given that untreated cases of TTP have a mortality rate of up to 95%, any clinical suspicion of TTP should prompt rapid initiation of treatment [1].

Similar to other autoimmune conditions, treatment is based on removing circulating antibodies and restoring proper levels of ADAMTS13. This is accomplished by TPE in the acute setting, as well as intravenous (IV) steroids in cases that require it. Although patients may present with severe anemia and thrombocytopenia, the transfusion of red blood cells and platelets is contraindicated before treating the underlying etiology. Circulating antibodies can attack the transfused blood products causing worsening of the condition. Refractory cases that don’t respond to TPE or steroids can benefit from rituximab. Relapsing and refractory cases have also been treated with immunosuppressive therapies such as vincristine, cyclophosphamide, cyclosporine A, or even splenectomy. Response to treatment is measured mainly by a symptomatic response as well as levels of lactate dehydrogenase (LDH), schistocytes on blood smears, and platelets. Levels of ADAMTS13 are usually measured at follow-up. Patients who receive prompt treatment with TPE have a very favorable prognosis, with an average survival rate of ~90% [2].

Rituximab is a monoclonal antibody against CD20. CD20 is a surface marker found on B cells, a type of white blood cell responsible for making antibodies. The thought behind using rituximab in this setting is that it attacks the source of antibody production.

## Case presentation

A 42-year-old female with no significant past medical history presented to the ER with a chief complaint of AMS. On arrival, the patient was confused and agitated and required sedation with Lorazepam and Haloperidol. History was provided by the family at the bedside who stated that for the past three to four weeks, the patient had been experiencing generalized malaise, bruising, headaches, chest pain, and abdominal pain. On the day of admission, the patient had chest pain with shortness of breath (SOB). There were no reported fevers or sick contacts for the patient.

Our patient had no previous medical history, surgical history, and no family history of blood disorders. She was married and lived with her husband; the patient took no medications, did not smoke, drink alcohol, or do illicit drugs.

On arrival, the patient had normal temperature, blood pressure 130/77 mmHg, pulse 115 beats per minute, and 20 respirations per minute. On physical exam, she was very confused and agitated. Multiple ecchymoses were noted but the exam was otherwise negative; the neurological exam was non-focal. The patient was noted to have gross hematuria. Labs showed platelets of 13000, hemoglobin 9.9, white blood cells (WBC) 11200, LDH 1669, and bilirubin 2.8. Blood urea nitrogen (BUN) was 14 while creatinine was 0.9 and potassium 3.2. At this time, suspicion for TTP was very high and the patient was transferred to the ICU and started on daily TPE. To rule out infection, computed tomography (CT) head, blood cultures, and spinal tap were performed; all with negative results.

The next morning, the patient’s WBC was 8400, hemoglobin 8.4, platelets 8000, and bilirubin 5.6. The diagnosis at that point was beginning to look more like TTP than anything else. The patient’s overall condition didn’t improve dramatically with the first TPE treatment, but gross hematuria did resolve. ADAMTS13 levels were measured and found to be undetectable during this hospital admission. After recovering during the first hospital admission, the patient was switched to twice weekly TPE, followed by once weekly, and was finally tapered off completely.

The patient relapsed two years later, requiring hospitalization and treatment with TPE. She was also started on rituximab once weekly for a total of four weeks. The next relapse was the following year and was treated with TPE daily for six doses and discharged on 60 mg prednisone, which was tapered off eventually. Five months later, the patient relapsed again and was again treated with TPE for a total of five treatments and rituximab for a total of four treatments. After this treatment, the patient’s ADAMTS13 was detectable for the first time at 34%.

The most recent and last relapse for our patient was over a year ago, which was treated with five treatments of TPE and four of rituximab. After this treatment, her ADAMTS13 level was 46%. Of note, the patient’s ADAMTS13 levels were always undetectable at the times of relapse. Given the improvement after rituximab treatment and the rise in ADAMTS13 levels, it was hypothesized that the patient might benefit from maintenance rituximab therapy. Rituximab every two months was initiated and the patient has not had a relapse in over a year. The ADAMTS13 level rose to 60% earlier this year, only one month after starting the treatment; the most recent level was 65%. The patient has been symptom and relapse-free with no deficiencies in complete blood count (CBC) since starting maintenance rituximab; this to go along with a consistent rise in ADAMTS13 levels.

## Discussion

There are currently no set guidelines for the chronic use of rituximab as maintenance therapy for TTP. Therapy is empirically based, from treatment in the acute setting to reestablish normal CBC parameters and to improve patient symptomatology. Rituximab demonstrates effectiveness in cases of TTP that are refractory. The addition of rituximab to TPE can accelerate recovery.

Patients with autoimmune disorders, such as rheumatoid arthritis, inflammatory bowel disease, and pemphigus vulgaris, have traditionally benefited from the use of rituximab. Similar therapeutic mechanisms in autoimmune diseases explain why rituximab predisposes patients to infection. By extinguishing B cells, rituximab decreases the levels of circulating antibodies, resulting in immunosuppression [3]. While this can reduce disease severity, immunosuppression can lead to infections and even reactivation of chronic infections such as hepatitis. Infusion reactions in certain patients can occur; otherwise, rituximab is usually well-tolerated. Given certain side effects, it cannot be used chronically [4].

While there are case reports demonstrating success with rituximab to prevent the relapse of TTP, there are no guidelines for treatment. The present case suggests a regimen with good efficacy and minimal side effects that may serve as a template for such a guideline. Continued research will be needed to establish a guideline for treatment [5]. The efficacy of this treatment regimen does offer hope for patients who suffer from TTP and the distress of recurrence.

## Conclusions

Scheduled dosing of rituximab can increase the level of ADAMTS 13 and reduce the rate of TTP relapse and hospitalization. This chronic scheduled infusion of rituximab also induces and sustains higher levels of ADAMTS13, which were previously undetectable. Our case evinces that a regimen of rituximab infusion every two months sustains CBC parameters within normal limits. More data will be needed to establish treatment guidelines.
